# Roles of Two-Dimensional Materials in Antibiofilm Applications: Recent Developments and Prospects

**DOI:** 10.3390/ph17070950

**Published:** 2024-07-16

**Authors:** Lei Xin, Hongkun Zhao, Min Peng, Yuanjie Zhu

**Affiliations:** 1 Department of Ultraasound, Naval Medical Center, Naval Medical University, Shanghai 200052, China; 2 Outpatient Department, Naval Medical Center, Naval Medical University, Shanghai 200052, China; 3 Department of Dermatology, Naval Medical Center, Naval Medical University, Shanghai 200052, China

**Keywords:** biofilm, two-dimensional nanomaterials, MoS_2_, MXene, black phosphorus, photothermal therapy (PTT), photodynamic therapy (PDT)

## Abstract

Biofilm-associated infections pose a significant challenge in healthcare, constituting 80% of bacterial infections and often leading to persistent, chronic conditions. Conventional antibiotics struggle with efficacy against these infections due to the high tolerance and resistance induced by bacterial biofilm barriers. Two-dimensional nanomaterials, such as those from the graphene family, boron nitride, molybdenum disulfide (MoS_2_), MXene, and black phosphorus, hold immense potential for combating biofilms. These nanomaterial-based antimicrobial strategies are novel tools that show promise in overcoming resistant bacteria and stubborn biofilms, with the ability to circumvent existing drug resistance mechanisms. This review comprehensively summarizes recent developments in two-dimensional nanomaterials, as both therapeutics and nanocarriers for precision antibiotic delivery, with a specific focus on nanoplatforms coupled with photothermal/photodynamic therapy in the elimination of bacteria and penetrating and/or ablating biofilm. This review offers important insight into recent advances and current limitations of current antibacterial nanotherapeutic approaches, together with a discussion on future developments in the field, for the overall benefit of public health.

## 1. Introduction

Antimicrobial resistance, particularly stemming from biofilm formation, represents a prominent hurdle in treating bacterial infections. While antibiotics represent the primary therapeutic approach for bacterial infections [1], their overuse has fostered antimicrobial resistance, undermining the efficacy of traditional antibiotic treatments and fueling the proliferation of drug-resistant pathogens [2,3]. Notably, the presence of biofilm significantly complicates the clearance of bacteria at infection sites.

Biofilm represents clustered communities of bacteria that adhere firmly to both living and non-living surfaces and are encapsulated in a secreted extracellular polymeric substance (EPS). This EPS, a complex assembly of exopolysaccharides, extracellular DNA, proteins, and enzymes, provides a shelter for planktonic microbes, promoting their survival, growth, and communication through quorum sensing [4,5,6,7]. While antibiotic treatment can usually alleviate symptoms caused by free-living bacteria, eliminating bacteria entrenched within biofilms and the biofilms themselves poses a formidable challenge due to their substantially heightened antibiotic resistance, ranging from 10 to 1000 times greater than that of planktonic bacteria [8,9].

Several factors contribute to this increased drug resistance. Firstly, many bacteria within biofilms are dormant and insensitive to antibiotics. Secondly, EPS acts as a barrier, preventing antibiotic penetration and shielding against attacks from the host’s innate immune system [10,11]. Unfortunately, incomplete biofilm removal often results in recurrence of the infection, systemic infections, and even therapeutic failures. Therefore, the effective elimination of stubborn biofilms is of great importance in the achievement of favorable outcomes when treating bacterial infections.

Graphene was the first two-dimensional material to be synthesized and has attracted extensive attention from the scientific community because of its many excellent properties [12,13,14,15,16]. In recent years, research and development on graphene have aroused renewed interest [17]. Although there is currently no clear definition of two-dimensional materials, several conditions are recognized by scientists, namely, that the structure of the material is in an ordered state, with a shape formed along the two-dimensional plane and a thin third dimension. It is generally believed that there is complete exposure of the surface atoms of two-dimensional materials in the single-layer formation, which greatly improves the utilization rate of the atoms. By controlling the thickness of the material, together with the doping of various elements, it is also easy to modulate the band gap and chemical properties. The advantages of two-dimensional materials lie in their extensive specific surface areas, controllable electrical conductivity, high atomic density on the exposed surface, controllable self-assembly and abundant active sites on their surfaces [18,19,20,21]. Two-dimensional nanomaterials include the graphene family, boron-nitride, molybdenum disulfide (MoS_2_), MXene, and black phosphorus. Due to their specific chemical and crystal structures, the properties of two-dimensional materials differ not only from those of graphene, but are also significantly different from each other, leading to significant potential applications in fields such as catalysis, biomedicine, energy storage, and nanoelectronics [22,23,24].

**Scheme 1:**
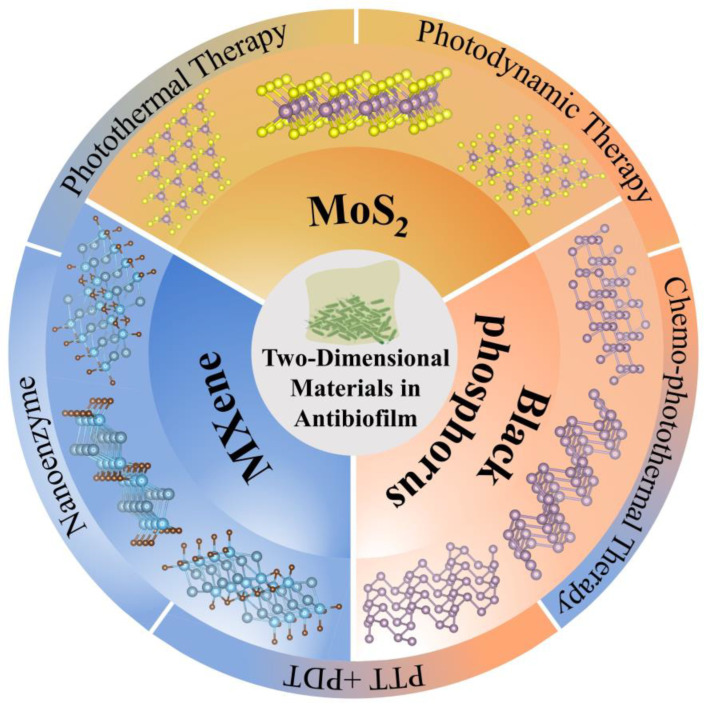
Two-dimensional nanomaterials for antibiofilm.

Faced with the difficulty of removing biofilms, together with the expense and difficulties associated with the development of new drugs, researchers are focusing on the exploration of novel approaches for the elimination of biofilms. Of these, two-dimensional nanomaterial-based approaches are being developed as investigated as antimicrobial and antibiofilm treatments. These include antimicrobial polymers, photothermal therapy (PTT), and photodynamic therapy (PDT), as well as nanomaterial applications for targeted delivery and drug release for killing bacteria [25]. The present review comprehensively summarizes recent advances in the use of two-dimensional nanomaterials in antibiofilm therapy, as well as a discussion of their antibacterial design, preparation, efficacies, both *in vitro* and *in vivo*, and treatment mechanisms, with examples (Scheme 1), which are compiled as follows (Table 1). Given the extensive and in-depth research in the graphene field, this will not be covered in this review. This review of promising antimicrobial and antibiofilm strategies for nanomaterials will inspire further development in the field and ultimately benefit public health.

## 2. MoS_2_ for Antibiofilm Applications

Recently, transition metal dichalcogenides (TMDs) were recognized for their atomic-scale thickness and useful attributes, making them appealing for a range of applications [46]. Among these, the semiconductor material MoS_2_ shows minimal cytotoxicity and has valuable physicochemical properties, rendering it suitable for a wide array of opto-electrochemical uses [47]. Depending on the conditions in which they were synthesized, MoS_2_ nanomaterials vary in both structure and properties, making them highly versatile for different applications. MoS_2_ and MoS_2_-based nanocomposites are particularly promising as antimicrobial materials due to their unique physicochemical attributes [48]. Moreover, MoS_2_ is becoming more widely used in biomedicine, primarily due to its excellent biocompatibility, which minimizes damage to normal tissue [49].

### 2.1. Carrier

In terms of carrier systems, Jie Zhao and colleagues successfully designed and prepared a novel antimicrobial system that combined mechano-bactericidal (Au-nanostars) and photothermal (MoS_2_) mechanisms [26]. Additionally, the surface modification in MoS_2_–Au nanocomposites with the targeting molecule vancomycin (Van-MoS_2_–Au) enhanced their antibacterial efficiency, allowing for effective action within specific radii. These nanocomposites demonstrated the ability to completely eradicate both Gram-negative and Gram-positive bacteria (*E. coli* and *B. subtilis,* respectively) using 20 min of 808 nm near-infrared (NIR) laser irradiation, with minimal bacterial proliferation seen even after 12 h. Furthermore, they showed efficacy in destroying refractory biofilms, presenting a significant advancement in addressing challenging medical issues. These novel antibacterial nanomaterials hold promise for various biomedical applications given their biocompatibility and potent antibacterial capabilities.

### 2.2. PDT

Alberto Escarpa and colleagues introduced a fuel-free approach for eliminating biofilm produced by *E. coli* and *S. aureus* using WS_2_ and MoS_2_ photophoretic microflakes [27]. These were fabricated by subjecting the materials to liquid-phase exfoliation. Following 480 or 535 nm irradiation, the microflakes exhibited rapid swarming at speeds exceeding 300 µm s^−1^ driven by photophoresis. Concurrently, the irradiation led to the production of reactive oxygen species (ROSs). The swift motion of the microflakes, forming multiple moving swarms, created an effective “collision” strategy that disrupted the biofilm structure, facilitating contact between the ROS and the bacteria, and leading to the inactivation of the latter. Consequently, impressive biofilm mass removal rates exceeding 90% and 65% were found when using MoS_2_ and WS_2_ microflakes against *E. coli* and *S*. *aureus* biofilms, respectively, within 20 min. In static conditions, significantly lower removal rates (30%) were observed, underscoring the importance of microflake mobility and ROS production in actively eradicating biofilms. Comparatively, the efficacy of biofilm deactivation using moving microflakes far surpasses that of free antibiotics, which struggle to penetrate densely packed biofilms. This innovative approach holds significant promise for combating antibiotic-resistant bacteria.

### 2.3. PTT

Jie Liu and colleagues designed a nanosystem, AA@Ru@HA-MoS_2_, using chemo-PTT (CPTT) for the treatment of bacterial infections [28]. This innovative system comprised mesoporous ruthenium nanoparticles (Ru NPs) as the nanocarrier, loaded with the prodrug ascorbic acid (AA) and surrounded by hyaluronic acid (HA). MoS_2_, pre-coated with ciprofloxacin, was used as a catalyst interacting with the outer surface. Upon accumulation at the infection site, the nanosystem underwent degradation of the HA capping by bacterial-secreted Hyal, triggering the release of AA. This release, coupled with catalysis by MoS_2_, led to the in situ generation of hydroxyl radicals (•OH). Additionally, leveraging the effective photothermal properties of Ru NPs, the nanosystem enabled combined CPTT. Demonstrating potent bactericidal activity against a variety of drug-resistant bacteria, the nanosystem also displayed efficacy in biofilm breakdown, inhibition of contained bacteria, and prevention of new biofilm formation.

### 2.4. Combined PTT and PDT

**Figure 1:**
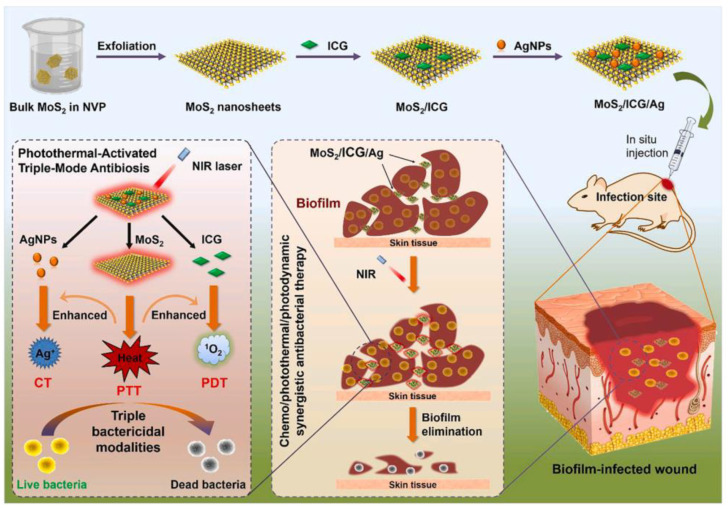
Schematic illustration for the preparation of multifunctional MoS_2_/ICG/Ag nanocomposites for the photothermally activated triple-mode synergistic antibacterial therapy. Copyright 2022, Elsevier.

Combined therapy holds promise for enhancing treatment efficacy and reducing side effects, making it a valuable approach for addressing bacterial infections, particularly those involving biofilms. Dinggeng He and collaborators proposed a PT-activated multifunctional platform, achieved by incorporating indocyanine green (ICG) photosensitizers and silver nanoparticles (AgNPs) onto the surface of MoS_2_ nanosheets [29]. The platform, MoS_2_/ICG/Ag, as shown in Figure 1, was effective against bacteria under a single 808 nm laser irradiation. Hyperthermia produced by the MoS_2_ could eradicate bacteria directly, as well as promote the release of ICG and silver ions to enhance the effect. The ICG can catalyze oxygen conversion to singlet oxygen when irradiated at 808 nm while acting synergistically with AgNPs to generate heat. The MoS_2_/ICG/Ag was effective against a wide variety of bacteria, while significantly inhibiting the development of *S. aureus* biofilms and effectively eradicating bacteria deep within the biofilms.

Fei Ge and colleagues designed an integrated photothermal therapy (PTT)/photodynamic therapy (PDT)/nitric oxide (NO) nanoplatform (MSC@CaCO_3_) [30]. The MoS_2_ nanospheres were first prepared, followed by the attachment of sodium nitroxide alginate (SANO) and dihydroporphyrin e6 (Ce6) to enable the release of NO and ROS when irradiated. Calcium carbonate is sensitive to acid and thus, when encountering the acidic environment of the biofilm, it cracks and releases drugs, with free Ca^2+^ assisting the SA in promoting wound healing. The photothermal conversion efficiency was found to be 48.9%, together with over 96% inhibition of multidrug-resistant *E. coli* and *S. aureus*. Additionally, MSC@CaCO_3_ effectively inhibited the proliferation of drug-resistant biofilms.

### 2.5. Nanozymes

MoS_2_ is a well-known photothermal agent in the antibacterial field, offering good biocompatibility. However, its therapeutic efficacy for deep tissue infections is limited by its restricted tissue penetration ability in the near-infrared (NIR-I) region and its reliance on a single treatment mode. Jianliang Shen and collaborators addressed this challenge by fabricating a hybrid 2H-MoS_2_ nanozyme (MoWS_2_) using a hydrothermal method [31]. This novel system combined antimicrobial activity with photothermal and enzymatic catalysis, with enhanced nanozyme activity achieved through controlled overheating. The introduction of tungsten ions to modulate 2H-MoS_2_ defects improved both NIR two-region (NIR-II) and enzymatic performances. *In vitro* experiments demonstrated that MoWS_2_ effectively killed bacteria and cleared biofilm through hyperthermia and ROS generation, as well as eradicating deep MRSA tissue infections via PTT and chemodynamic therapy (CDT). These effects were verified in histological and immunofluorescence analyses, highlighting its potential for the efficient treatment of deep tissue infections.

**Figure 2:**
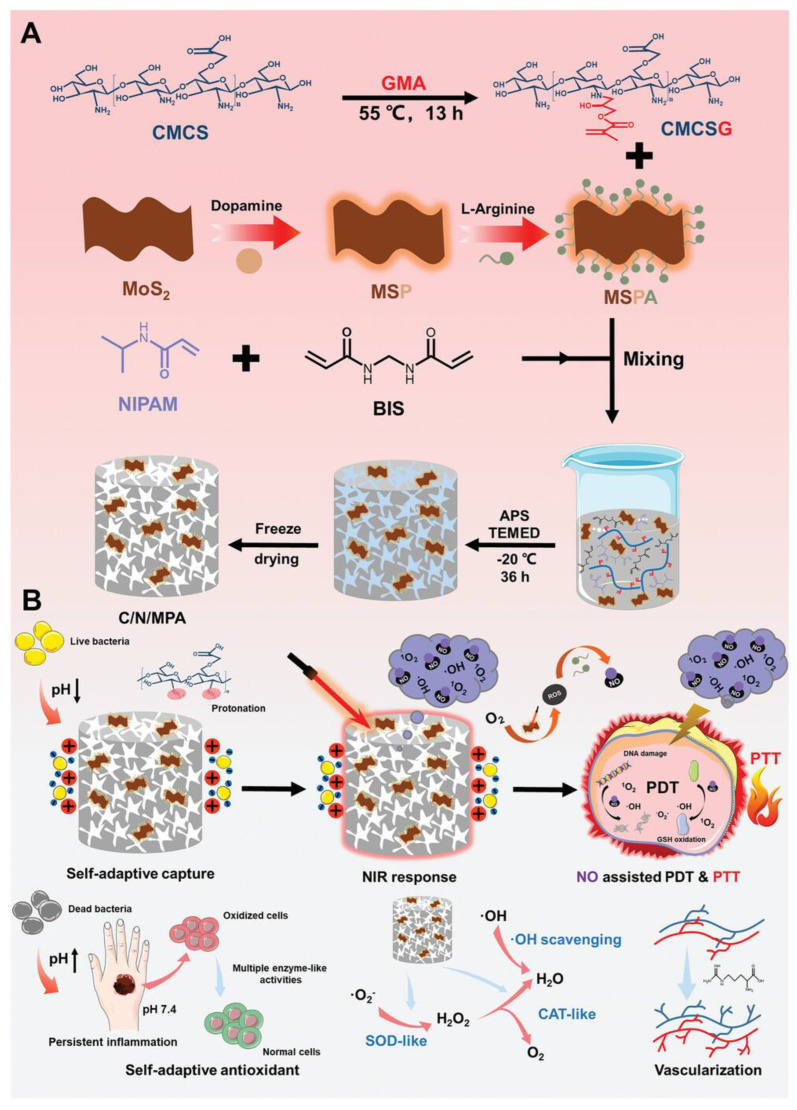
Schematic illustration of the preparation and application of multiple stimuli-responsive nanozyme-based cryogel. (**A**) The preparation of CMCSG, MSPA and C/N/MPA; (**B**) Mechanism of multiple stimuli-responsive antibacterial and self-adaptive wound management. Copyright 2023, Wiley-VCH.

Baolin Guo and colleagues introduced wound dressings that could respond to multiple stimuli and eliminate bacteria [32]. MoS_2_, together with L-arginine that could release NO upon sensing ROS, was incorporated into carboxymethyl chitosan/poly(N-isopropylacrylamide)-based cryogels (CMCS/PNIPAM) that could react to pH, NIR, and temperature changes, thus being able to dynamically adjust to different stages and requirements in the healing of infected wounds (Figure 2). In the moderately acidic microenvironments associated with bacterial infections, microbial capture was facilitated through protonation, thus enhancing the photodynamic antibactericidal effects. The application of NIR light promoted the release of ROS and NO. These cryogels were found to eradicate MRSA biofilms using NO-assisted PDT and PTT.

## 3. MXenes for Antibiofilm Applications

Among the diverse array of 2D nanomaterials, the transition metal carbide/nitride/carbonitride (MXene) family has emerged prominently over the last decade [50,51]. MXenes constitute the largest family of 2D materials, of which more than 30 different types have been described and many more investigated computationally. These materials are derived from MAX phases, where the A-layer is selectively removed to expose the first MXene layer, resulting in 2D flakes. MAX phases consist of multilayer hexagonal structures, with M and X representing the components of MXenes and A indicating an A-group element. The weak M-X bond allows for the selective etching of an atom wedged between them.

MXenes exhibit atomically thin thickness but potentially limitless lateral dimensions, with surface atoms offering remarkable characteristics that captivate material scientists and nanotechnology developers. They boast superior conductivity and mechanical properties, as well as unique plasmonic, optical, and thermoelectric attributes, and are widely used in a variety of fields, including energy storage, electronics, catalysis, electrochemical and electromagnetic applications, sensing, and biomedicine [52,53,54].

Recent years have witnessed a burgeoning interest in biomedical applications of MXene, such as cancer treatments (e.g., photothermal therapy), theranostics, bioimaging, drug delivery, biosensing, and antibacterial treatment [55,56,57,58,59]. Nevertheless, studies on antimicrobial properties remain limited, with only a few demonstrating MXene nanosheets’ ability to eliminate bacteria by interacting directly with the cell membranes, inducing oxidative stress [60,61]. Additionally, research on the interaction between MXene and biofilms remains insufficient. The following provides a summary of the current literature in this field.

### 3.1. Unmodified or Modified MXenes

To enhance its antibacterial performance, MXene can be conjugated with other nanomaterials. Jianping Xie and colleagues developed a synergistic antimicrobial agent by conjugating ultra-small gold nanoclusters (AuNCs) onto MXene nanosheets [33]. This conjugation allows for the effective delivery of AuNCs into bacteria following MXene-induced membrane damage, resulting in the generation of localized ROSs that effectively oxidize bacterial membrane lipids and DNA. Synergy between physical (through MXene) and chemical (through MXene and AuNCs) antibacterial effects can kill a variety of bacteria, with low IC_50_ values. Moreover, the crumpled structure of the MXene-AuNCs inhibits biofilm formation, combining MXene-AuNCs’ synergistic antibacterial ability with surface hydrophobicity preventing bacterial adhesion and high densities of bactericides.

**Figure 3:**
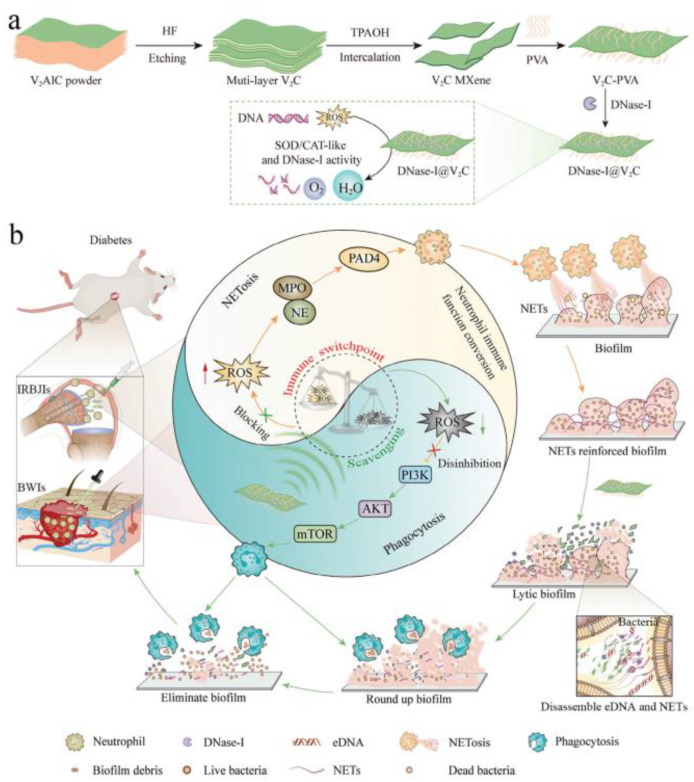
Schematic describing (**a**) the design and synthetic process of DNase-I@V_2_C and (**b**) the neutrophil immune function conversion (NIFC) mechanism of DNase-I@V_2_C in combating diabetic-related biofilm infections. IRBJIs, implant-related biofilm joint infections; BWIs, biofilm wound infections. Copyright 2023, Wiley-VCH.

Diabetes-related biofilm infections (DRBIs) pose challenges due to their well-developed biofilms and dysregulation of neutrophils resulting from oxidative stress. Hao Shen and collaborators proposed an approach involving immune activation of neutrophils using a DNase I-loaded vanadium carbide MXene (DNase-I@V_2_C) [34] (Figure 3). The DNase-I@V_2_C was effective in scavenging ROS in environments associated high levels of oxidative stress, thus preserving the DNase I activity. As DNase-I@V_2_C was able to penetrate deeply into the biofilm, it could degrade extracellular DNA and NETs, thus disrupting biofilm structure. Moreover, through its function in regulating immune switch points, DNase-I@V_2_C could redirect neutrophil function toward phagocytosis by inhibition of ROS–NE/MPO–PAD_4_ and activation of the ROS–PI3K–AKT–mTOR axis, thus breaking down the biofilm. This strategy has demonstrated promising efficacy, indicating the potential of the modulation of immune switch points to influence neutrophil activities and thus treat DRBIs.

### 3.2. PTT

In an effort to harness the therapeutic possibilities of vanadium, Xin Pan and collaborators developed a vanadium-MXene nanosheet, 4K10@V_2_C, by coating a four-armed peptidomimetic involved in host defense, 4K10, electrostatically onto vanadium carbide (V_2_C) MXene [35]. This two-dimensional system had photothermal ability, and was able to induce membrane breakdown and photocatalysis, thus presenting significant promise for efficiently targeting focal infections via a microneedle-supported approach. The novel strategy of augmenting MXene with the host defense peptidomimetic induced synergistic photothermal and membranolytic actions against biofilm bacteria by selective binding, decreasing bacterial resistance to heat, and promoting heat transfer from the MXene to the bacteria. Additionally, the degradation products of V_2_C were able to prevent bacterial regrowth through long-lasting photocatalytic disinfection. *In vitro* studies demonstrated the impressive antimicrobial activity of 4K10@V_2_C nanosheets, with a remarkable decrease in methicillin-resistant *S. aureus* (MRSA) by approximately 99.9996% (5.5 log) and complete eradication of *Pseudomonas aeruginosa* at the relatively low temperature of 54.1 °C. Furthermore, the microneedle system, which subsequently dissolved, enhanced the delivery of the 4K10@V_2_C nanosheets across the skin, surpassing the efficacy of commercial Bactroban without significant toxicity. The nanosheets were also effective for eliminating biofilm infections in ex vivo models of human skin infections, with a reduction in skin MRSA burden exceeding 99.98% (3.86 log). This study highlighted the potential of 4K10@V_2_C nanosheets as a highly effective and safe therapeutic agent for combating biofilm-associated infections.

### 3.3. Combined PTT and PDT

**Figure 4:**
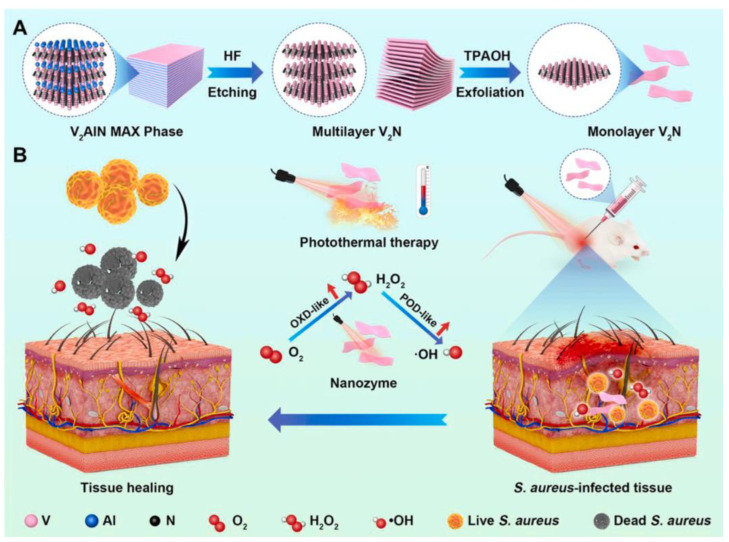
Schematic illustration of (**A**) the preparation of monolayer V_2_N and (**B**) 1064 nm laser irradiation enhanced dual-enzyme-like catalytic activities of V_2_N for promoting the healing of S. aureus-infected abscesses *in vivo*. Copyright 2023, Elsevier.

Nanocatalytic technology holds great promise for treating infections through the use of synthetic nanoscale enzyme mimics known as nanozymes. However, the activity of nanozymes can often be limited by reliance on a single catalytic function in the complex microenvironment of the infected tissue. Addressing this challenge, Rongdang Hu and collaborators designed a vanadium nitride MXene (V_2_N) through a chemical exfoliation method [36] (Figure 4). The unique properties of V_2_N, including its ability to switch valency and its large surface area, provided both oxidase- and peroxidase-like enzymatic activities in weakly acidic environments similar to those of biofilm. Additionally, V_2_N exhibited favorable photothermal conversion efficiency (PCE) in the NIR-II region, thus enhancing its catalytic activity while reducing the likelihood of thermal damage to normal tissues in the vicinity. These combined attributes enabled ROS generation, resulting in the effective eradication of bacteria *in vitro* and V_2_N to generate abundant ROS, effectively eradicating bacteria and promoting subcutaneous abscess healing with minimal toxicity. This innovative approach, using photothermal promotion of enzymatic activities, based on photothermal-enhanced dual-enzyme-like catalytic activities, holds promise in the treatment of infections.

### 3.4. Nanozymes

Despite the development of various approaches leveraging ROS, many still suffer from inefficiency. Lu-Lu Qu and colleagues synthesized Ag-MXene nanozymes with peroxidase-like activities for treating infections [37]. The Ag-MXene nanozyme efficiently elevates intracellular ROS levels through the conversion of H_2_O_2_ into hydroxyl radicals, effectively eradicating a variety of bacteria and their biofilms. Ag-MXene was also used to develop a colorimetric biosensor for assaying cholesterol, relying on its peroxidase-like activity. Demonstrating high performance, the biosensor was effective for detecting cholesterol in the 2–800 μM range with a detection limit of 0.6 μM. Notably, Ag-MXene nanozymes enable rapid cholesterol detection in serum without the need for complex sample pretreatment. Overall, the proposed Ag-MXene nanozymes hold promise as both biocides and cholesterol sensors, offering broad prospects for both assays and sterilization in the biomedical field.

## 4. Black Phosphorus for Antibiofilm Applications

Black phosphorus (BP) has the greatest stability of all phosphorus varieties [62]. BP has garnered significant attention in recent years for its diverse applications in biomedicine, including bioimaging, PTT, PDT, biosensing, drug delivery, and more [63,64]. In BP, the phosphorus atoms are interconnected by chemical bonding, while van der Waals forces hold the layers together [65]. The band gap of BP varies according to the layer thicknesses, allowing for modulation from 0.3 eV overall to 2.0 eV for an individual layer. This characteristic leads to strong light absorption across a wide range of visible light spectra, distinguishing BP from other 2D materials [66,67,68]. The antimicrobial properties, excellent biocompatibility, and biodegradability of BP make it suitable for various applications in biomedicine. Recent studies have demonstrated the exceptional PDT performance of BP nanosheets (BPSs) in cancer treatment, attributed to their high singlet oxygen generation ability and good biocompatibility [69]. However, the instability of BP poses a challenge for its biomedical applications. Extensive efforts have been devoted to enhancing the stability of BP through methods such as surface coordination, tellurium doping, chemical modification, and noncovalent functionalization [70,71,72].

### 4.1. PTT

Biofilm infections pose a significant challenge for medical implants, often leading to progressive tissue destruction and systemic diseases. Wang Lin and colleagues devised an antimicrobial photothermal system by combining polydopamine (PDA)-BP nanosheets (BP NSs) with ZnO nanowires (NWs) on Ti substrates to tackle these concerns [38]. The combination of BP NSs and PDA resulted in superior PTT ability upon NIR irradiation that, notably, not only eliminated biofilm but also promoted Zn^2+^ release from ZnO, significantly enhancing its antibacterial efficacy. The localized PTT effectively dispersed bacterial biofilms, while ZnO NWs thoroughly eliminated bacteria within the biofilms *in vitro*, showcasing enhanced sterilization capabilities.

Infections of the urinary tract caused by catheters and similar devices have long been a concern due to their adverse effects on medical equipment usage and patient health [73,74,75]. Therefore, it is imperative to develop catheters from biocompatible materials with antibacterial capabilities. Di Huang and colleagues fabricated polylactic acid (PLA)-based electrospun membranes that incorporated BP nanosheets (BPNS) and nano-zinc oxide (nZnO), individually and in combination, to produce active membranes with antibacterial properties [39]. The ZnO-BP/PLA membranes had porous structures with uniformly distributed nZnO and BPNS, offering improved mechanical properties with increased polylactic acid concentration. Additionally, the membranes also showed significant PTT abilities when exposed to NIR, resulting in the breakdown of the biofilms and promoting Zn^2+^ release. Cytotoxicity and adhesion experiments revealed good cytocompatibility, with normal cell growth on the ZnO-BP/PLA antibacterial membrane surface.

The engineering of multifunctional bionic coatings on the surfaces of implants is gaining traction. Guo Xiang and colleagues loaded 2D BP nanosheets (BPs) onto metal implants coated with hydroxyapatite (HA) for the construction of a BPs@HA composite coating [40]. Due to its PCE and biomineralization *in situ*, the coating was found to be highly effective in the eradication of biofilm and promoting healing of the fracture.

Wei Zhao and colleagues developed a rhamnolipid-BP nanocomposite (RHL@BP) for treating drug-resistant *H. pylori* (*H. pylori*-R) [41]. This was used to deliver isolinderalactone (ISL), a sesquiterpenoid derived from traditional Chinese medicinal herbs, which markedly enhanced its antimicrobial effectiveness against *H. pylori*-R when irradiated with NIR light. The BP sensitivity to acid, together with the photothermal effects of RHL induced ISL release, showed marked antibacterial actions.

Imren Hatay Patir and colleagues were the first to investigate the photothermal effects of BP/Au nanocomposites following NIR irradiation [42]. This involved the synthesis of BP crystals by chemical vapor transport and their exfoliation onto BP nanosheets in hexane or deoxygenated water. Dispersed Au nanoparticles were synthesized and assembled on the nanosheets in hexane, yielding BP/Au nanocomposites. Due to their superior PCE relative to the BP nanosheets alone, they were able, after NIR irradiation, to disrupt microbial cell membranes, resulting in a 58% reduction in biofilm.

### 4.2. PDT

Rong Wang and colleagues explored the use of *Elaeagnus mollis* polysaccharide (EMP) in modifying BP to enhance its bactericidal activity against foodborne pathogens [43]. The EMP-BP composite was found to be more stable and effective relative to either EMP or BP alone. This was the result of photocatalysis generating ROS and membrane disruption by the polysaccharides, leading to cell death. Moreover, EMP-BP reduced both biofilm development and the expression of virulence factors in *S. aureus*, in addition to showing good biocompatibility in hemolysis and cytotoxicity assessments. Additionally, the EMP-BP-treated bacteria remained sensitive to antibiotics and did not develop resistance.

**Figure 5:**
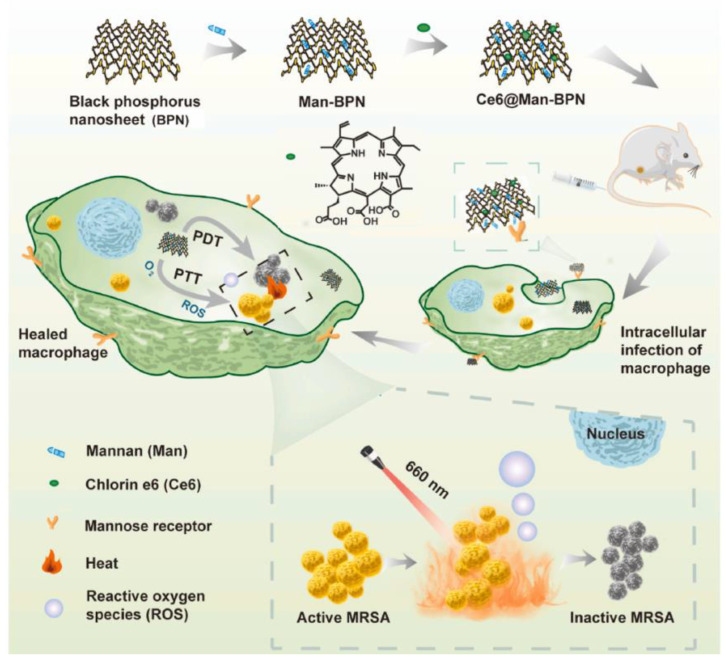
Synthesis and targeting of Ce6@Man-BPN to macrophages and demonstration of the inactivation of intracellular bacteria by combined photothermal and photodynamic therapy. Copyright 2023, Elsevier.

Intracellular infections caused by resistant bacteria are a significant problem. Novel systems capable of precise targeting and antimicrobial efficacy are highly sought after for combating MRSA within macrophages. Wenjun Miao and co-researchers introduced a groundbreaking photoactive nanoplatform for targeted elimination of intracellular pathogens. They utilized mannosylated BP nanosheets (Man-BPN) as carriers loaded with chlorin e6 (Ce6), resulting in Ce6@Man-BPN, as shown in Figure 5. This formulation targeted macrophages through selective recognition of mannose receptors on the infected cells. Ce6@Man-BPN effectively eliminated intracellular MRSA through a combined PTT/PDD with minimal cytotoxicity. This approach presents a promising avenue for *in vivo* biofilm clearance.

### 4.3. Chemo-Photothermal Therapy (CPTT)

**Figure 6:**
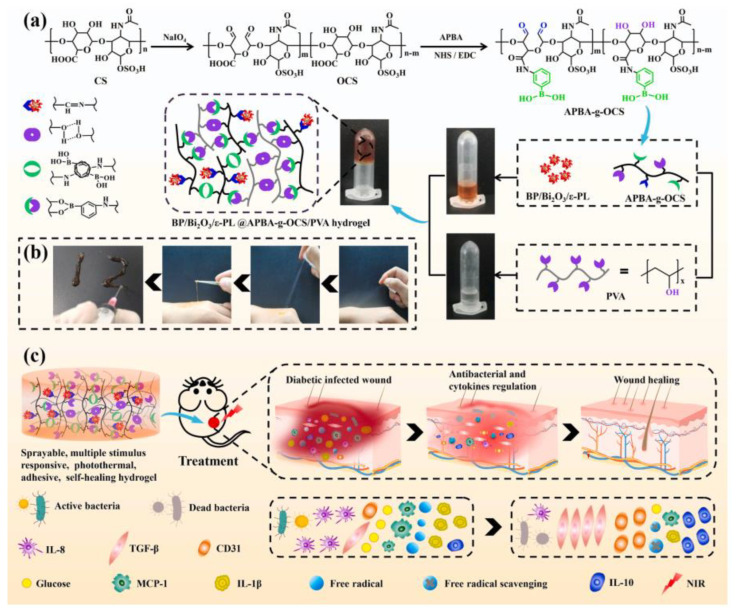
Preparation of the hydrogels: (**a**) schematic illustration of OCS, APBA-G-OCS and NPs@gel-2; (**b**) injectable and sprayable process of NPs@gel-2; (**c**) schematic diagram of the hydrogel that promotes healing of infected diabetic wounds by combined chemo-photothermal antimicrobial and cytokines modulation. Copyright 2023, Elsevier.

To tackle the challenge of healing diabetic foot (DF) wounds, often complicated by resistant microbial biofilms and wound microenvironments, Cui Cheng developed a multifunctional hydrogel [45]. This hydrogel, prepared via in situ or spray methods, incorporated oxidized chondroitin sulfate modified by 3-aminophenylboronic acid (APBA-g-OCS), BP/bismuth oxide/ε-polylysine (BP/Bi_2_O_3_/ε-PL), and polyvinyl alcohol (PVA) as precursors to facilitate the healing of diabetic wounds, as shown in Figure 6. These hydrogels exhibited responsiveness to a variety of stimuli, together with robust adhesion and the ability to self-heal rapidly due to the presence of borate ester bonding, hydrogen bonding, and π–π interactions, resulting in effective crosslinking. They also demonstrated synergistic CPTT antimicrobial activities and the ability to break down biofilm resulting from the incorporation of BP/Bi_2_O_3_/ε-PL via dynamic imine bond cross-linking. Additionally, they possessed antioxidant and anti-inflammatory effects due to their ability to adsorb chemokines, attributed to the APBA-g-OCS. Crucially, these hydrogels were not only able to react to the microenvironment of the wound to induce both PTT and chemotherapy, thus reducing inflammation, but could also treat the microenvironment of the wound by scavenging ROS and modulating the levels of cytokines. This dual functionality accelerated collagen deposition and promoted both angiogenesis and the formation of granulation tissue and angiogenesis, ultimately facilitating the healing of infected diabetic wounds in experimental rat models.

## 5. Conclusions and Future Outlook

Recent years have seen the emergence of MoS_2_ nanosheets as promising agents for NIR light–thermal conversion. While MoS_2_ has shown some potential as a bactericidal agent [76], its development and application are still in the early stages. Several challenges have slowed the progress of MoS_2_-based antibacterial nanomaterials. For instance, their antimicrobial efficacy has been diminished by the relative limited interactions with the bacteria and the short distances involved in the interactions, thus falling short of the prerequisites for effective antimicrobial performance. Moreover, the inherent antimicrobial capability of MoS_2_ nanosheets is constrained, necessitating the development of synergistic bactericidal platforms to enhance efficacy. Additionally, understanding the mechanism of action of MoS_2_ against biofilms remains crucial. Despite the reported nanostructured MoS_2_ variants, their biomedical applications and environmental impacts require further exploration, with a particular emphasis on rigorous quality control to ensure safety and efficacy.

Exploring antibiofilm therapeutics using two-dimensional nanomaterial-based approaches, which are integral in treating biofilm-related infections, reveals several pertinent limitations. Firstly, ensuring biocompatibility and safety is paramount as two-dimensional nanomaterials may provoke immune or toxic responses in living organisms [77,78,79]. Rigorous *in vitro* and *in vivo* toxicity assessments are imperative before advancing to clinical trials. Secondly, addressing stability and persistence is crucial, as biological factors can compromise the stability and durability of two-dimensional nanomaterials within living organisms. Efforts should focus on enhancing their stability to maintain therapeutic efficacy [47]. Lastly, achieving targeted delivery and penetration into complex biological environments where biofilm infections occur is essential. Strategies to precisely target infection sites and penetrate biofilms are necessary to achieve therapeutic goals effectively. To facilitate the clinical translation of effective antibiofilm two-dimensional nanomaterials, a comprehensive evaluation and understanding of the contributions made by the structures and compositions of the specific nanomaterials to the eradication of bacteria and biofilms is essential. This understanding will aid in the optimization of both the chemical and structural properties of the nanomaterials, paving the way for a further rational design and synthesis of the next-generation nanoplatforms.

The two primary challenges in the application of two-dimensional nanomaterials for antibiofilm purposes are as follows: Firstly, there is the technological hurdle of achieving large-scale production, considering the intricacies of the exfoliation process and the challenges in scaling up from the laboratory to industrial levels. Secondly, while two-dimensional nanomaterials exhibit their antibiofilm properties due to their single-atomic-layer thickness, they tend to aggregate in practical applications, altering their properties and thereby compromising their antibiofilm efficacy. Consequently, future research must delve deeper into exfoliation mechanisms, optimize production processes, and develop innovative stabilizers to foster the efficient and stable utilization of two-dimensional nanomaterials in the antibiofilm domain.

It is crucial to acknowledge that the use of two-dimensional nanomaterials in the treatment and elimination of biofilm is primarily at the level of academic research and thus requires preclinical validation before advancing to clinical application. There are a number of essential issues, including the mechanisms underlying the antibiofilm activities of the different platforms and the metabolic pathways involved, that require comprehensive investigation. Additionally, critical considerations regarding translation to the clinical sphere, including cost effectiveness, scalability, safety concerns, product reproducibility, and long-term environmental effects, must be carefully addressed.

## Figures and Tables

**Table 1:** Overview of two-dimensional nanomaterials for antibiofilm.

Type of Nanomaterials	Bactericidal Agents	Bacteria	References
MoS_2_	Van-MoS_2_–Au	*E. coli* and *B. subtilis*	[26]
	MoS_2_ and WS_2_ microflakes	*E. coli* and *S. aureus*	[27]
	AA@Ru@HA-MoS_2_	MDR *S. aureus* and MDR *P. aeruginosa*	[28]
	MoS_2_/ICG/Ag	*E. coli* and *S. aureus*	[29]
	MSC@CaCO_3_	MDR *E. coli* and MDR *S. aureus*	[30]
	MoWS_2_	MRSA	[31]
	C/N/MPA cryogel	*E. coli* and MRSA	[32]
MXene	MXene-AuNCs	*E. coli* and *S. aureus*	[33]
	DNase-I@V_2_C	*S. aureus*	[34]
	K10@V_2_C	MRSA and *P. aeruginosa*	[35]
	V_2_N	*S. aureus* and *S. mutans*	[36]
	Ag-MXene	*E. coli* and *S. aureus*	[37]
Black phosphorus	Ti-PDA/BP/ZnO	*E. coli* and *S. aureus*	[38]
	ZnO-BP/PLA	*E. coli* and *S. aureus*	[39]
	BPs@HA	*E. coli* and *S. aureus*	[40]
	RHL@BP/ISL	*H. pylori*	[41]
	BP/Au	*E. faecalis*	[42]
	EMP-BP	*S. aureus*	[43]
	Ce6@Dex-BPN	MRSA	[44]
	BP/Bi_2_O_3_/ε-PL	*E. coli*, *S. aureus* and *P. aeruginosa*	[45]

## Data Availability

No new data were created or analyzed in this study. Data sharing is not applicable to this article.
